# Soret and Dufour effects on a Casson nanofluid flow past a deformable cylinder with variable characteristics and Arrhenius activation energy

**DOI:** 10.1038/s41598-021-98898-6

**Published:** 2021-09-29

**Authors:** Naila Shaheen, Hashim M. Alshehri, Muhammad Ramzan, Zahir Shah, Poom Kumam

**Affiliations:** 1 Department of Computer Science, Bahria University, Islamabad, 44000 Pakistan; 2 Department of Mathematics, Faculty of Science, King Abdulaziz University, Jeddah, 21521 Saudi Arabia; 3 Department of Mathematical Sciences, University of Lakki Marwat, Lakki Marwat, 28420 Khyber Pakhtunkhwa Pakistan; 4 Fixed Point Research Laboratory, Fixed Point Theory and Applications Research Group, Center of Excellence in Theoretical and Computational Science (TaCS-CoE), Faculty of Science, King Mongkut’s University of Technology Thonburi (KMUTT), 126 Pracha Uthit Rd., Bang Mod, Thung Khru, Bangkok, 10140 Thailand; 5 Department of Medical Research, China Medical University Hospital, China Medical University, Taichung, 40402 Taiwan

**Keywords:** Mechanical engineering, Software

## Abstract

In this study, the effects of variable characteristics amalgamated with chemical reaction and Arrhenius activation energy are analyzed on a two-dimensional (2D) electrically conducting radiative Casson nanoliquid flow past a deformable cylinder embedded in a porous medium. The surface of the cylinder is deformable in the radial direction i.e*.,* the *z-*axis. The impression of Soret and Dufour's effects boosts the transmission of heat and mass. The flow is analyzed numerically with the combined impacts of momentum slip, convective heat, and mass conditions. A numerical solution for the system of the differential equations is attained by employing the bvp4c function in MATLAB. The dimensionless protuberant parameters are graphically illustrated and discussed for the involved profiles. It is perceived that on escalating the velocity slip parameter and porosity parameter velocity field depreciates. Also, on escalating the radiation parameter and heat transfer Biot number a prominent difference is noticed in an upsurge of the thermal field. For growing values of Brownian motion and thermophoretic parameters, temperature field augments. On escalating the curvature parameter and porosity parameter, drag force coefficient upsurges. The outcome of the Soret number, mass transfer Biot number, and activation energy parameter is quite eminent on the concentration distribution for the sheet in comparison to the deformable cylinder. A comparative analysis of the present investigation with an already published work is also added to substantiate the envisioned problem.

## Introduction

Researchers have immensely emphasized the transmission of heat past an elongated surface. It has enormous applications in the process of manufacturing, engineering, and industries such as continuous stretching of plastic sheets, aerodynamics, hot rolling, paper production, cooling of metallic plates, bar drawing, extrusion, and glass blowing. A numerical solution for time-independent two-phase Casson nanoliquid flow past a horizontal elongated cylinder is presented by Ramzan et al.^1^. The flow is incorporated with variable heat source/sink and Newtonian heating. It is found that the thermal field escalates for both phases for rising values of Newtonian heating. Reddy et al.^2^ discussed the stagnation point flow of a radiative Eyring Powell liquid flow across an inclined stretchable cylinder. Buongiorno model is considered. Fluid flow is enhanced with an additional effect of Cattaneo Christov (CC) heat flux and chemical reaction. It is concluded that thermal and solutal field uplifts for rising values of curvature parameter. The upshot of homogeneous-heterogeneous reaction on an incompressible nanoliquid flow across a stretchable chamber is elucidated analytically and numerically by Sankar et al.^3^. It is observed that the thermal field boosts on amplifying the curvature parameter and Hartmann number. Mishra et al.^4^ interpreted the influence of viscous dissipation combined with Ohmic dissipation on spherical and cylindrical-shaped nanoparticles over an elongated horizontal cylinder. It is witnessed that fluid flow accelerates for cylindrically shaped nanoparticles, however, a reverse trend is exhibited for spherical shaped nanoparticles. Consequently, exploration in this regard with different physical aspects can be seen in^5–15^.

In the fluid flow, two mechanisms are involved in the conduction of heat. First, when the collision amid the molecules increases. Second, thermal conductivity plays a key role in escalating the random movement among the molecules. Thermal conductivity has significant applications in steam generators, electrolytes, concrete heating, and laminating. Abdelmalek et al.^16^ numerically analyzed second-order velocity slip and bio convection on a pseudoplastic nanoliquid flow over a deformable cylinder. In this study transmission of heat and mass is communicated with the addition of temperature-dependent thermal conductivity and activation energy. The aftermath of temperature-dependent thermal conductivity and chemical reaction on a radiative Williamson nanoliquid is notified by Ibrahim et al.^17^ past a horizontal stretchable cylinder immersed in a porous medium. The flow is incorporated with viscous dissipation and robin conditions. On a two-dimensional 2D radiative Non-Newtonian fluid flow past an extendable chamber is analytically demarcated by Raza et al.^18^ alongside the influence of variable molecular diffusivity and temperature-dependent thermal conductivity. The Buongiorno model is utilized here. It is contemplated that thermal field enhances on mounting the Eckert number and radiation parameter, whereas, a decreasing output is noticed for fluid velocity on boosting the Reynold number. On a time-dependent, Maxwell fluid Khan et al.^19^ numerically analyzed Cattaneo- Christov (CC) model over a deformable cylinder and sheet with variable thermal conductivity and mass diffusion. Further investigation on variable thermal conductivity across an elongated cylinder is mentioned in^20–25^.

The Soret-Dufour factor plays a key role in the transmission of heat and mass on a moving fluid. It has a vital role in several applications which include the design of nuclear reactors, geothermal energy, groundwater pollutant migration, oil reservoirs, isotopes separation, manufacture of rubber and plastic sheets, the mixture of gases, compact heat insulation exchanger, and nuclear waste disposal. The features of the Soret and Dufour effect amalgamated with the chemical reaction on an MHD couple stress liquid are analytically addressed by Gajjela et al.^26^ over an elongated cylinder. The findings disclosed that for growing values of curvature parameter thermal and solutal gradient upsurges. Tlili and Waqas^27^ numerically analyzed the impact of bio convection and second-order slip on a radiative Oldroyd–B nanoliquid flow past a linear deformable cylinder. The flow is enhanced with the additional effect of zero mass flux and convective heat conditions. It is computed that fluid temperature upsurges by varying thermophoresis and curvature parameters. Radiative flux with Soret and Dufour effect on a second-grade fluid over an elongated cylinder is illustrated by Shojaei et al.^28^. It is perceived in this exploration that the solutal and thermal field tumbles on escalating the Schmidt number and Prandtl number. Using the Buongiorno model Jawad and Saeed^29^ analytically explored the significance of the Soret Dufour factor on Maxwell fluid over a permeable elongated surface. The flow is incorporated with the addition of motile microorganisms and temperature-dependent thermal conductivity. It is reported that on mounting the Soret factor solutal field diminishes. Consequently, exploration in this regard with different physical aspects can be seen in refs.^30–43^.

Researchers have manifested concern about fluid flow across the permeable surface. The flow through the porous chamber is very common and have widespread applications in industries, natural circumstances, petroleum, and chemical engineering for instance crude oil extraction, storage of nuclear waste material, movement of oil and water across the oil reservoir, heat exchangers, drying process, MHD generators, thermal insulation, seepage of water in river beds, filtration and water purification process. Singh et al.^44^ investigated the combined influence of melting heat and variable heat source/sink with porosity effect across a horizontal stretchable cylinder. A numerical solution is obtained using the Keller box method. It is concluded that transmission of heat hikes on escalating the melting parameter, whereas, shear drag force diminishes for rising values of Reynold number. Bisht et al.^45^ numerically studied the transmission of heat for Sisko nanoliquid in a porous medium across a linear deformable cylinder. It is found that transfer of heat declines on boosting the Brownian and porosity parameter, however, an uplift is noticed for the curvature parameter. Thermal features on a hybrid nanoliquid are analytically discussed by Saeed et al.^46^ across a permeable elongated chamber. It is reported that fluid flow upsurges for rising values of curvature parameter. A reverse trend is observed in escalating the porosity parameter. The convective flow of hybrid nanoliquid in a porous medium through an elongated chamber is numerically explored by Aminian et al.^47^. It is revealed that enhancing the Hartman number rate of heat transfer escalates. Subsequently, exploration in this regard with different physical aspects can be seen in refs.^48–57^.

According to the above-mentioned literature review, most of the researchers have investigated the characteristics of thermal radiation and activation energy past an elongated cylinder. The purpose of the present investigation is to examine the effect of temperature-dependent thermal conductivity, variable mass diffusion on a radiative Casson nanofluid flow past a deformable cylinder. The impression of the Soret and Dufour effect boosts the transmission of heat and mass. The flow is analyzed numerically with the combined impact of heat generation/absorption, chemical reaction with activation energy, momentum slip, and robin condition. The mathematical model is deciphered through MATLAB software bvp4c. The outcome of numerous parameters is examined for the deformable cylinder and stretching sheet via tabular and graphical illustrations. The uniqueness of the presented mathematical model is illustrated in Table 1 by associating it with the published studies.

**Table 1:** An inspection of literature for the innovation of the presented model.

Authors	Stretching cylinder	Soret Dufour effect	Temperature-dependent thermal conductivity	Thermal radiation	Variable molecular diffusivity	Porous medium	Activation energy
Reddy et al.^2^	Yes	No	No	Yes	No	No	Yes
Abdelmalek et al.^16^	Yes	No	Yes	No	No	No	Yes
Tulu et al.^21^	Yes	No	Yes	No	No	Yes	No
Tlili et al.^27^	Yes	No	No	Yes	No	No	Yes
Shojaei et al.^28^	Yes	Yes	No	Yes	No	No	No
Jagan et al.^42^	Yes	Yes	No	Yes	No	No	No
Present	Yes	Yes	Yes	Yes	Yes	Yes	Yes

### Mathematical problem formulation

**Figure 1:**
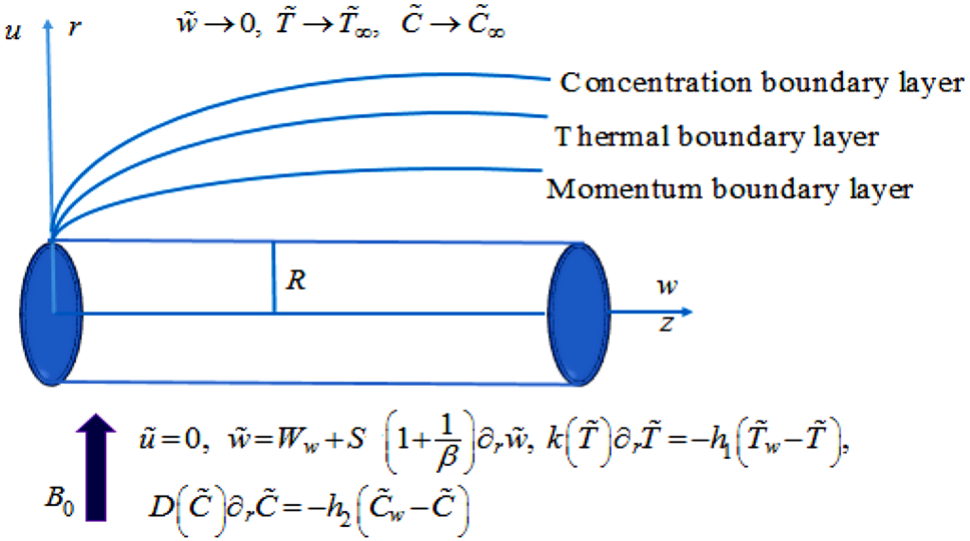
Flow representation of the model.

An incompressible, time-independent, 2D electrically conducting radiative Casson nanoliquid flow is examined past a deformable cylinder in a permeable medium. The nanoliquid model describes the attributes of Brownian motion and thermophoresis. The geometry of the problem is illustrated in cylindrical coordinate in such a manner that the cylinder is stretchable horizontally in the axial direction (i.e*., z-*axis) and the radial direction (i.e*., r-*axis) is orthogonal to it (Fig. 1). Transfer of heat and mass is enhanced with temperature-dependent thermal conductivity and variable molecular diffusivity incorporated with Soret and Dufour effect. Moreover, the impression of chemical reaction with activation energy, velocity slip effect, and robin conditions are analyzed.

The rheological equation for Casson fluid model is demarcated as^48,58^

1 $$ \tau_{ij} = \left\{ {\begin{array}{*{20}l} {\left( {\mu_{c} + \frac{{S_{y} }}{{\left( {2\tilde{\pi }} \right)^{0.5} }}} \right)2\tilde{\gamma }_{ij} ,} \hfill & {if \, \tilde{\pi } > \tilde{\pi }_{c} } \hfill \\ {\left( {\mu_{c} + \frac{{S_{y} }}{{\left( {2\tilde{\pi }_{c} } \right)^{0.5} }}} \right)2\tilde{\gamma }_{ij} ,} \hfill & {if \, \tilde{\pi } < \tilde{\pi }_{c} } \hfill \\ \end{array} } \right. $$

where $\tau_{ij}$ is the extra stress tensor and

2 $$ \begin{aligned} & \tilde{\pi } = \tilde{\gamma }_{ij} \tilde{\gamma }_{ij} \,\,{\text{is}}\,{\text{the}}\,{\text{product}}\,{\text{of}}\,{\text{the}}\,{\text{components}}\,{\text{of}}\,{\text{deformation}}\,{\text{rate}} \\ & \tilde{\gamma }_{ij} = \frac{1}{2}\left( {\partial_{{x_{j} }} v_{i} + \partial_{{x_{i} }} v_{j} } \right)\,\,{\text{is}}\,{\text{the}}\,{\text{rate}}\,{\text{of}}\,{\text{the}}\,{\text{strain}}\,{\text{tensor}} \\ & \tilde{\pi }_{c} \,\,{\text{is}}\,{\text{the}}\,{\text{critical}}\,{\text{value}}\,{\text{of}}\,{\text{deformation}}\,{\text{rate}}\,{\text{tensor}} \\ & S_{y} \,\,{\text{is}}\,{\text{the}}\,{\text{fluid}}\,{\text{yield}}\,{\text{stress}}. \\ \end{aligned} $$

The equations governing the flow^1,2,16,20,24^ are as follows:

3 $$ \partial_{r} \left( {r\tilde{u}} \right) + \partial_{z} \left( {r\tilde{w}} \right) = 0, $$

4 $$ \tilde{u}\partial_{r} \tilde{w} + \tilde{w}\partial_{z} \tilde{w} = \frac{\nu }{r}\left( {1 + \frac{1}{\beta }} \right)\partial_{r} \left( {r\partial_{r} \tilde{w}} \right) - \frac{{\sigma_{1} B_{0}^{2} }}{\rho }\tilde{w} - \frac{\nu }{{K^{*} }}\tilde{w}, $$

5 $$ \begin{aligned} \tilde{u}\partial_{r} \tilde{T} + \tilde{w}\partial_{z} \tilde{T} & = \frac{1}{{\rho c_{p} }}\frac{1}{r}\partial_{r} \left( {k\left( T \right)r\partial_{r} \tilde{T}} \right) + \tau \left( {D_{B} \partial_{r} \tilde{T}\partial_{r} \tilde{C} + \frac{{D_{T} }}{{\tilde{T}_{\infty } }}\left( {\partial_{r} \tilde{T}} \right)^{2} } \right) \\ & \quad - \frac{1}{{\rho c_{p} }}\frac{1}{r}\partial_{r} \left( {r\partial_{r} q_{r} } \right) + Q_{1} \frac{{\left( {\tilde{T} - \tilde{T}_{\infty } } \right)}}{{\rho c_{p} }} + \frac{{D_{T} k_{t}^{*} }}{{c_{s} c_{p} }}\frac{1}{r}\partial_{r} \left( {r\partial_{r} \tilde{C}} \right), \\ \end{aligned} $$

6 $$ \tilde{u}\partial_{r} \tilde{C} + \tilde{w}\partial_{z} \tilde{C} = \frac{1}{r}\partial_{r} \left( {D_{B} \left( {\tilde{C}} \right)r\partial_{r} \tilde{C}} \right) + \frac{{D_{T} k_{t}^{*} }}{{\tilde{T}_{\infty } }}\frac{1}{r}\partial_{r} \left( {r\partial_{r} \tilde{T}} \right) - k_{r}^{2} \left( {\frac{{\tilde{T}}}{{\tilde{T}_{\infty } }}} \right)\left( {\tilde{C} - \tilde{C}_{\infty } } \right)\exp \left( {\frac{{ - E_{a} }}{{k\tilde{T}}}} \right). $$

The mathematical form of radiative heat flux^2^ is as follows:

7 $$ q_{r} = - \frac{4}{3}\frac{{\overline{\sigma }}}{{\overline{k}}}\partial_{r} T^{4} ,\quad {\text{where }}T^{4} = 4T_{\infty }^{3} T - 3T_{\infty }^{4} $$

In Eq. (5), temperature-dependent thermal conductivity^20^ is stated as:

8 $$ k\left( T \right) = k_{\infty } \left( {1 + d\left( {\frac{{\tilde{T} - \tilde{T}_{{_{\infty } }} }}{{\tilde{T}_{w} - \tilde{T}_{\infty } }}} \right)} \right) $$

In Eq. (6), variable molecular diffusivity^20^ is expressed as:

9 $$ D_{B} \left( C \right) = D_{{B_{\infty } }} \left( {1 + e\left( {\frac{{\tilde{C} - \tilde{C}_{\infty } }}{{\tilde{C}_{w} - \tilde{C}_{\infty } }}} \right)} \right) $$

with boundary conditions^20,58^

10 $$ \begin{aligned} & \left. {\tilde{u}} \right|_{r = R} = 0,\,\,\left. {\tilde{w}} \right|_{r = R} = W_{w} + S\left( {1 + \frac{1}{\beta }} \right)\partial_{r} \tilde{w},\,\,\left. {k\left( T \right)\partial_{r} \tilde{T}} \right|_{r = R} = - h_{1} \left( {\tilde{T}_{w} - \tilde{T}} \right), \\ & \left. {D\left( C \right)\partial_{r} \tilde{C}} \right|_{r = R} = - h_{2} \left( {\tilde{C}_{w} - \tilde{C}} \right),\,\,\left. {\tilde{w}} \right|_{r \to \infty } \to 0,\,\,\left. {\tilde{T}} \right|_{r \to \infty } \to \tilde{T}_{\infty } ,\,\,\left. {\tilde{C}} \right|_{r \to \infty } \to \tilde{C}_{\infty } . \\ \end{aligned} $$

Using appropriate subsequent transformation^24,59^

11 $$ \begin{aligned} & \tilde{u} = - \frac{R}{r}.\left( {\frac{{\nu U_{1} }}{l}} \right)^{0.5} f\left( \zeta \right),\tilde{w} = \frac{{U_{1} z}}{l}f^{\prime}\left( \zeta \right),\zeta = - \left( {\frac{{U_{1} }}{l\nu }} \right)^{0.5} \left( {\frac{{R^{2} - r^{2} }}{2R}} \right), \\ & \theta \left( \zeta \right) = \frac{{\tilde{T} - \tilde{T}_{\infty } }}{{\tilde{T}_{w} - \tilde{T}_{\infty } }},\phi \left( \zeta \right) = \frac{{\tilde{C} - \tilde{C}_{\infty } }}{{\tilde{C}_{w} - \tilde{C}_{\infty } }}. \\ \end{aligned} $$

Utilizing transformation, Eq. (3) is trivially equated. Though Eqs. (4)–(6) and (10) are transmuted as:

12 $$ \left( {1 + \frac{1}{\beta }} \right)\left( {\left( {1 + 2\omega \zeta } \right)\frac{{d^{3} f}}{{d\zeta^{3} }} + 2\omega \frac{{d^{2} f}}{{d\zeta^{2} }}} \right) = f\frac{{d^{2} f}}{{d\zeta^{2} }} + \left( {\frac{df}{{d\zeta }}} \right)^{2} + \left( {Ha + \lambda } \right)\frac{df}{{d\zeta }}, $$

13 $$ \begin{aligned} & \left( {1 + 2\omega \zeta } \right)\left( {\left( {1 + d\theta } \right) + \frac{4}{3}Rd} \right)\frac{{d^{2} \theta }}{{d\zeta^{2} }} = - 2\omega \left( {\left( {1 + d\theta } \right) + \frac{4}{3}Rd} \right)\frac{d\theta }{{d\zeta }} \\ & \quad - \Pr \left( {f\frac{d\theta }{{d\zeta }} + D_{f} \left( \begin{gathered} 2\omega \frac{d\phi }{{d\zeta }} \hfill \\ + \left( {1 + 2\omega \zeta } \right)\frac{{d^{2} \phi }}{{d\zeta^{2} }} \hfill \\ \end{gathered} \right) + Q^{*} \theta } \right) - \left( {1 + 2\omega \zeta } \right)\left( \begin{gathered} d\left( {\frac{d\theta }{{d\zeta }}} \right)^{2} \hfill \\ + \Pr \left( {N_{b} \frac{d\theta }{{d\zeta }}\frac{d\phi }{{d\zeta }} + N_{t} } \right)\left( {\frac{d\theta }{{d\zeta }}} \right)^{2} \hfill \\ \end{gathered} \right), \\ \end{aligned} $$

14 $$ \left( {1 + e\phi } \right)\left( {1 + 2\omega \zeta } \right)\frac{{d^{2} \phi }}{{d\zeta^{2} }} = - \left( \begin{gathered} 2\omega \left( {1 + e\phi } \right) \hfill \\ + \left( {1 + 2\omega \zeta } \right)e\frac{d\phi }{{d\zeta }} \hfill \\ + S_{c} f \hfill \\ \end{gathered} \right)\frac{d\phi }{{d\zeta }} - S_{c} \left( \begin{gathered} S_{r} \left( \begin{gathered} 2\omega \frac{d\theta }{{d\zeta }} \hfill \\ + \left( {1 + 2\omega \zeta } \right)\frac{{d^{2} \theta }}{{d\zeta^{2} }} \hfill \\ \end{gathered} \right) \hfill \\ + \delta \phi \left( {1 + \alpha \theta } \right)\exp \left( {\frac{ - E}{{1 + \alpha \theta }}} \right) \hfill \\ \end{gathered} \right). $$

and the boundary conditions take the form:

$$ f\left( \zeta \right) = 0,\frac{df}{{d\zeta }} = 1 + L\left( {1 + \frac{1}{\beta }} \right)\frac{{d^{2} f}}{{d\zeta^{2} }},\frac{d\theta }{{d\zeta }} = - H_{1} \left( {\frac{1 - \theta \left( 0 \right)}{{1 + d\theta }}} \right), \, \frac{d\theta }{{d\zeta }} = - H_{2} \left( {\frac{1 - \phi \left( 0 \right)}{{1 + e\phi }}} \right){\text{ at }}\zeta { = 0} $$

15 $$ \frac{df}{{d\zeta }} \to 0, \, \frac{d\theta }{{d\zeta }} \to 0, \, \frac{d\phi }{{d\zeta }} \to 0{\text{ as }}\zeta \to \infty $$

The mathematical forms of shear stress at the wall (drag force coefficient), local Nusselt, and Sherwood number are specified as:

16 $$ C_{f} = \frac{{2\tau_{w} }}{{\rho W_{w}^{2} }}\quad \tau_{w} = \mu \left( {1 + \frac{1}{\beta }} \right)\left. {\partial_{r} \tilde{u}} \right|_{r = R} $$

17 $$ Nu_{z} = \frac{{zQ_{w} }}{{k_{\infty } \left( {\tilde{T}_{w} - \tilde{T}_{\infty } } \right)}}\quad \, Q_{w} = \left. { - k\left( T \right)\partial_{r} \tilde{T} + q_{r} } \right|_{r = R} $$

18 $$ Sh_{z} = \frac{{zQ_{m} }}{{D_{{B_{\infty } }} \left( {\tilde{C}_{w} - \tilde{C}_{\infty } } \right)}}\quad \, Q_{m} = \left. { - D_{B} \left( C \right)\partial_{r} \tilde{C}} \right|_{r = R} $$

By employing Eq. (11), the dimensionless form of Eq. (16)–(18) are as follow:

19 $$ \frac{1}{2}\left( {{\text{Re}}_{z} } \right)^{0.5} C_{f} = \left( {1 + \frac{1}{\beta }} \right)\left. {\frac{{d^{2} f}}{{d\zeta^{2} }}} \right|_{\zeta = 0} $$

20 $$ Nu_{z} \left( {{\text{Re}}_{z} } \right)^{ - 0.5} = - \left( {1 + \frac{4}{3}\left( {\frac{Rd}{{1 + d\theta }}} \right)} \right)\left. {\frac{d\theta }{{d\zeta }}} \right|_{\zeta = 0} $$

21 $$ Sh_{z} \left( {{\text{Re}}_{z} } \right)^{ - 0.5} = - \left( {1 + e\phi } \right)\left. {\frac{d\phi }{{d\zeta }}} \right|_{\zeta = 0} $$

## Numerical procedure

The solution of the system of highly nonlinear differential equations can be obtained by numerous analytical, exact, and numerical procedures^10,13,56,60–71^. The proposed mathematical model is handled numerically. Here, the coupled nonlinear ODEs are computed numerically by employing the bvp4c function in MATLAB. Mentioned numerical code is used, we obtain ODEs which are of order one.

22 $$ \begin{aligned} & f = Y_{1} ,f^{\prime} = Y_{2} ,f^{\prime\prime} = Y_{3} ,f^{\prime \prime \prime } = Y_{3}^{\prime } = YY_{1} , \\ & YY_{1} = \left( {\left( {\frac{1}{{1 + \frac{1}{\beta }}}} \right)\left( {\frac{1}{1 + 2\omega \zeta }} \right)} \right)\left( {Y_{1} .Y_{3} + Y_{2}^{2} + \left( {Ha + \lambda } \right)Y_{2} } \right) - \left( {\frac{2\omega }{{1 + 2\omega \zeta }}} \right)Y_{3} , \\ & \theta = Y_{4} ,\theta ^{\prime} = Y_{5} ,\theta ^{\prime\prime} = Y_{5}^{\prime } = YY_{2} , \\ & \phi = Y_{6} ,\phi ^{\prime} = Y_{7} ,\phi ^{\prime\prime} = Y_{7}^{\prime } = YY_{3} . \\ & YY_{2} = \left( {\frac{1}{{\left( {1 + 2\omega \zeta } \right)\left( {\left( {1 + d.Y_{4} } \right) + \frac{4}{3}Rd} \right)}}} \right)\left( \begin{gathered} - \left( {\left( {1 + 2\omega \zeta } \right)d.Y_{5} + 2\omega \left( {\left( {1 + d.Y_{4} } \right) + \frac{4}{3}Rd} \right)} \right)Y_{5} \hfill \\ - \Pr \left( {1 + 2\omega \zeta } \right)\left( {N_{b} Y_{5} .Y_{7} + N_{t} Y_{5}^{2} } \right) \hfill \\ - \Pr \left( {2\omega .D_{f} .Y_{7} + f.Y_{5} + Q^{*} .Y_{4} + D_{f} \left( {1 + 2\omega \zeta } \right).YY_{3} } \right) \hfill \\ \end{gathered} \right), \\ & YY_{3} = \left( {\frac{1}{{\left( {1 + e.Y_{6} } \right)\left( {1 + 2\omega \zeta } \right)}}} \right)\left( \begin{gathered} - \left( {e.Y_{7} .\left( {1 + 2\omega \zeta } \right) + 2\omega \left( {1 + e.Y_{6} } \right)} \right)Y_{7} \hfill \\ - S_{c} \left( \begin{gathered} Y_{1} .Y_{7} + S_{r} \left( {2\omega .Y_{5} + \left( {1 + 2\omega \zeta } \right).YY_{2} } \right) \hfill \\ + \delta .Y_{6} \left( {1 + \alpha .Y_{4} } \right)^{n} \exp \left( {\frac{ - E}{{1 + \alpha .Y_{4} }}} \right) \hfill \\ \end{gathered} \right) \hfill \\ \end{gathered} \right). \\ & {\text{and}}\,{\text{the}}\,{\text{boundary}}\,{\text{conditions}}\,{\text{are}}\,{\text{enumerated}}\,{\text{as}} \\ & Y_{1} (0) = 0,Y_{2} (0) = 1 + L\left( {1 + \frac{1}{\beta }} \right)Y_{3} \left( 0 \right),Y_{5} \left( 0 \right) = - H_{1} \left( {\frac{{1 - Y_{4} \left( 0 \right)}}{{1 + d.Y_{4} \left( 0 \right)}}} \right),Y_{7} \left( 0 \right) = - H_{2} \left( {\frac{{1 - Y_{6} \left( 0 \right)}}{{1 + e.Y_{6} \left( 0 \right)}}} \right),\quad {\text{at}}\quad \, \zeta { = 0} \\ & Y_{2} (\infty ) \to 0,Y_{4} (\infty ) \to 0,Y_{6} (\infty ) \to 0.{\text{ as }}\zeta \to \infty \\ \end{aligned} $$

## Analysis of results

**Figure 2:**
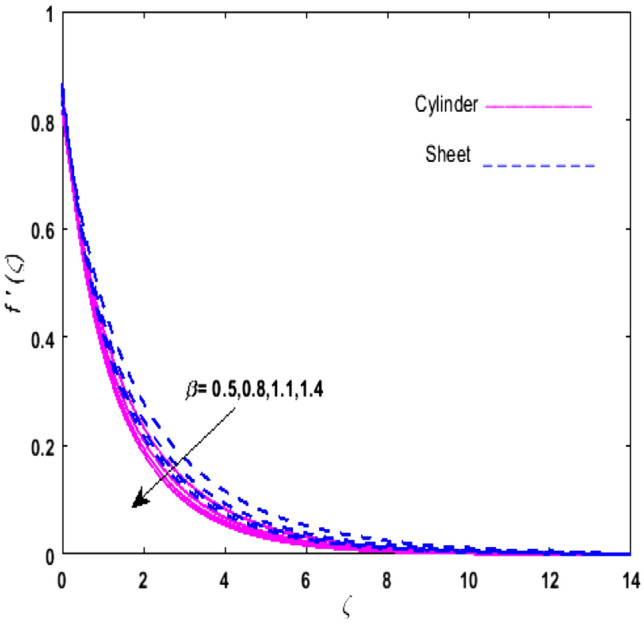
Upshot of $\beta$ on $f^{\prime}\left( \zeta \right)$.

**Figure 3:**
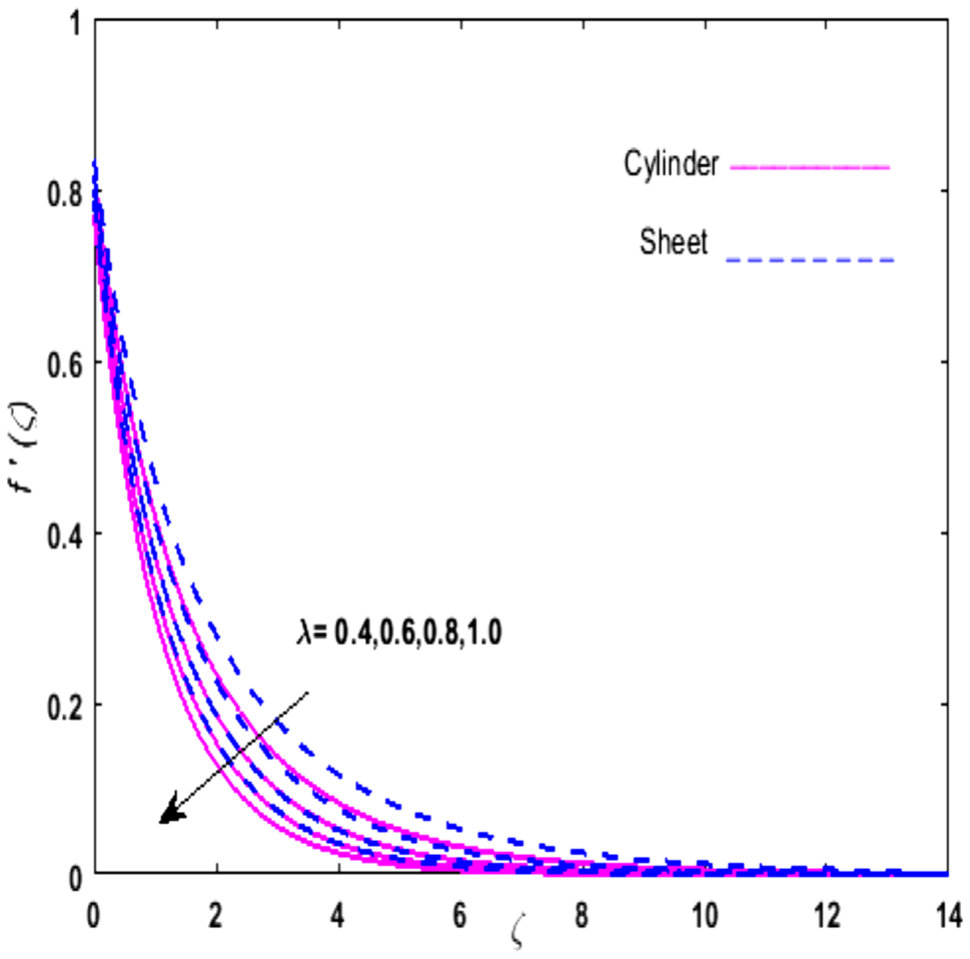
Upshot of $\lambda$ on $f^{\prime}\left( \zeta \right)$.

**Figure 4:**
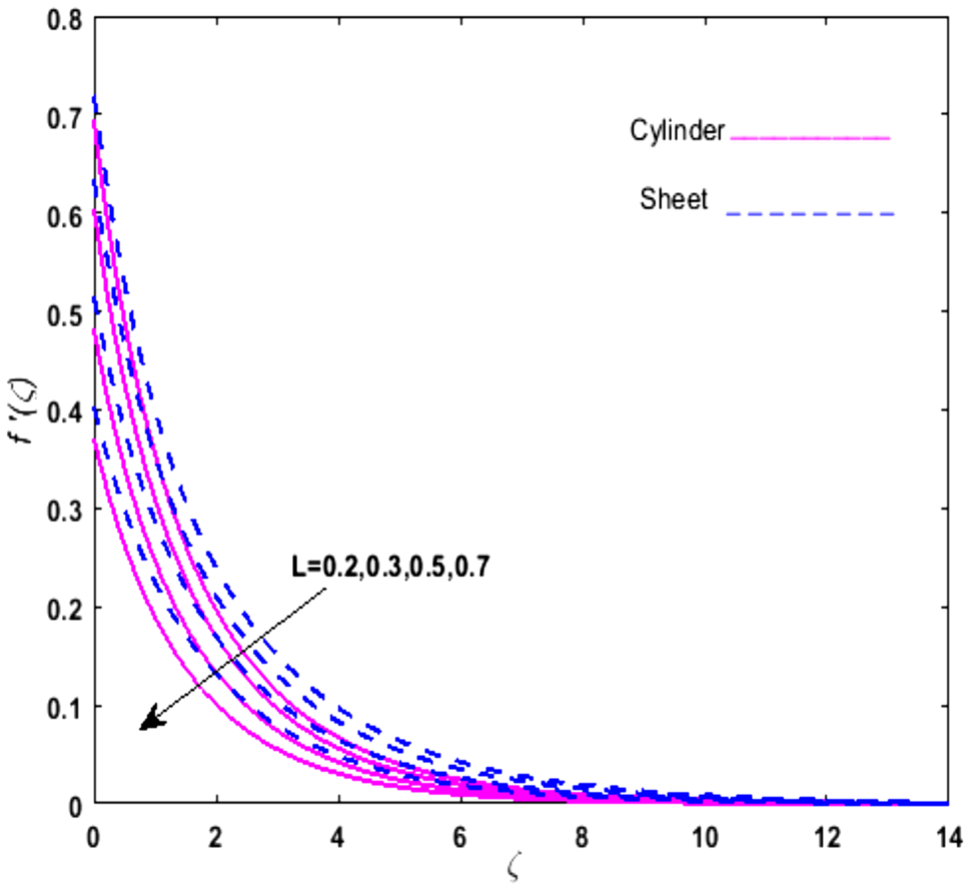
Upshot of $L$ on $f^{\prime}\left( \zeta \right)$.

**Figure 5:**
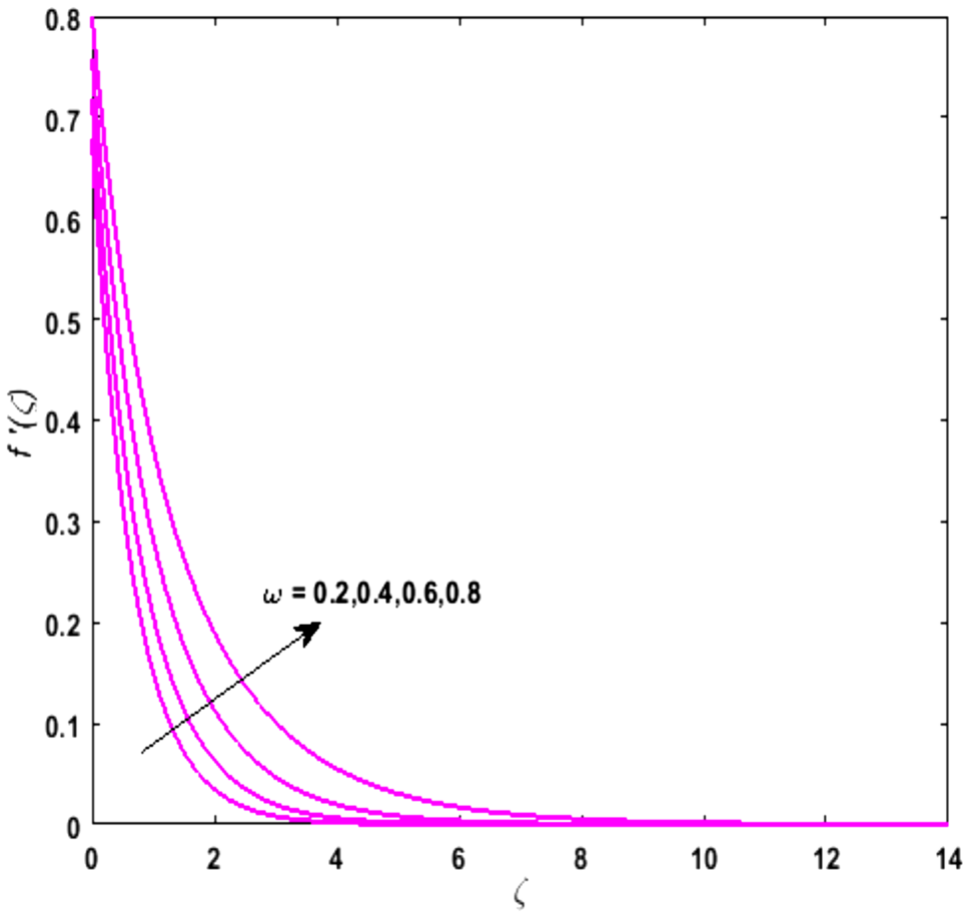
Upshot of $\omega$ on $f^{\prime}\left( \zeta \right)$.

**Figure 6:**
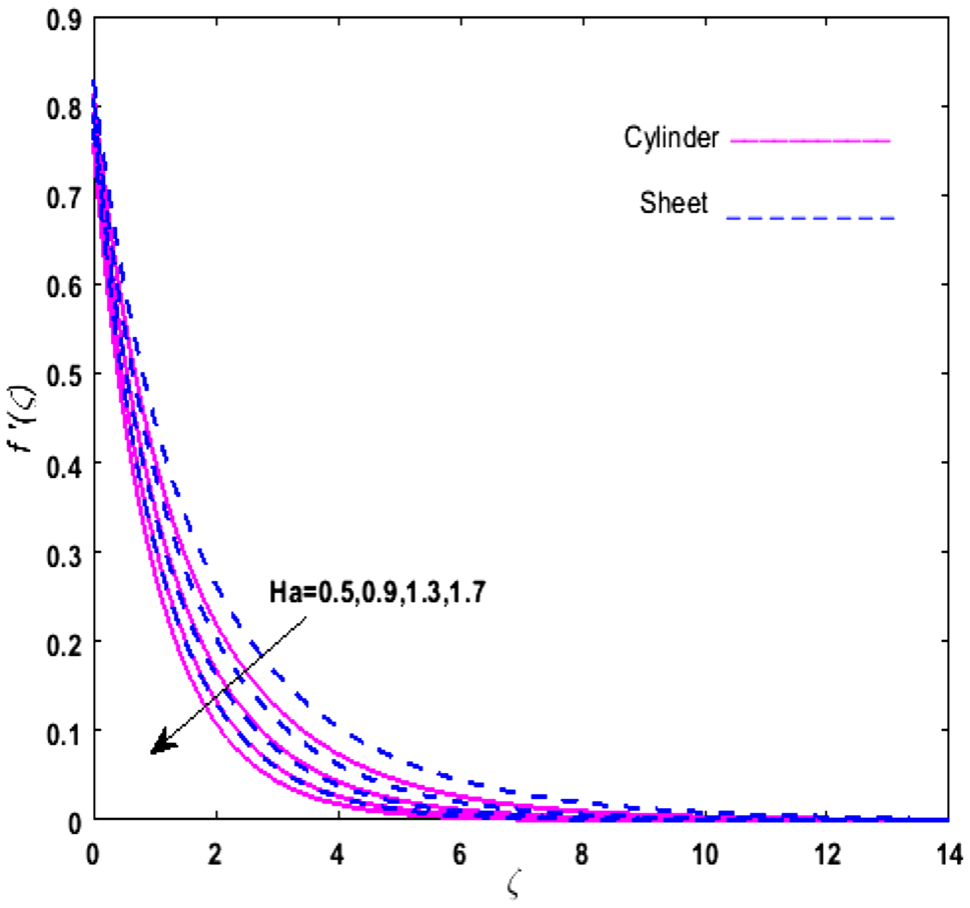
Upshot of $Ha$ on $f^{\prime}\left( \zeta \right)$.

**Figure 7:**
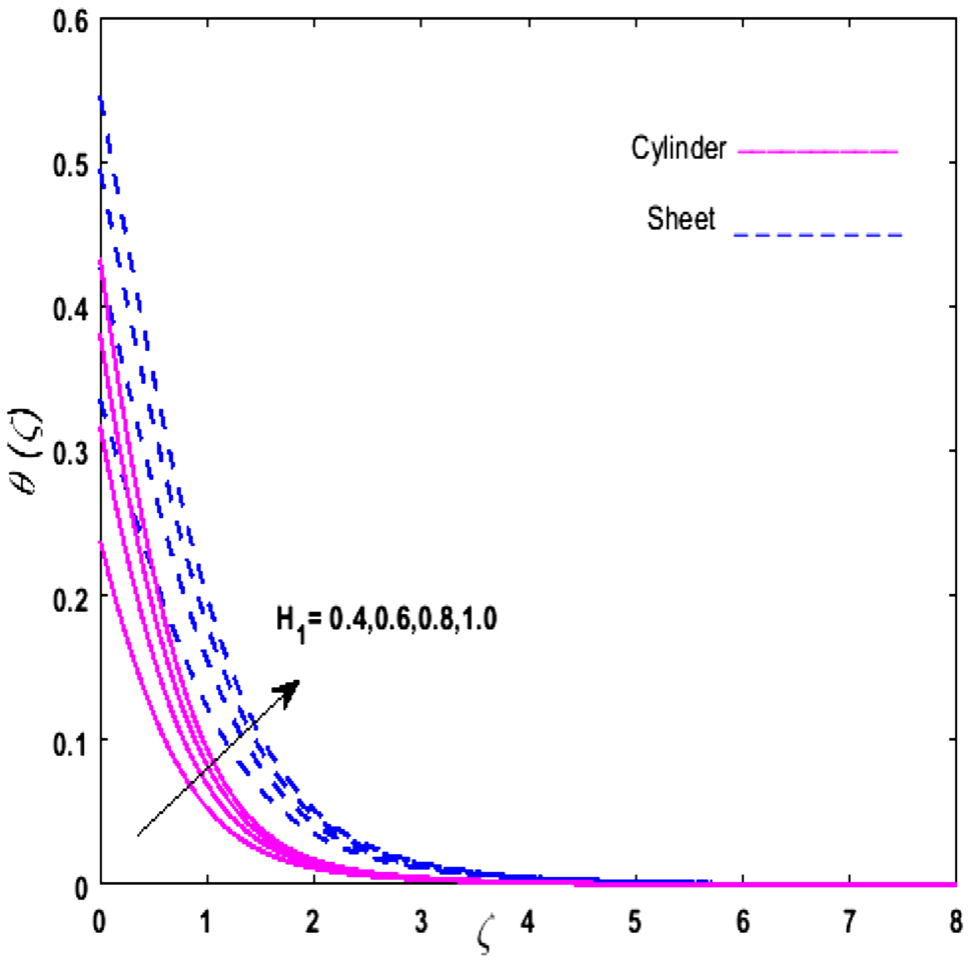
Upshot of $H_{1}$ on $\theta \left( \zeta \right)$.

**Figure 8:**
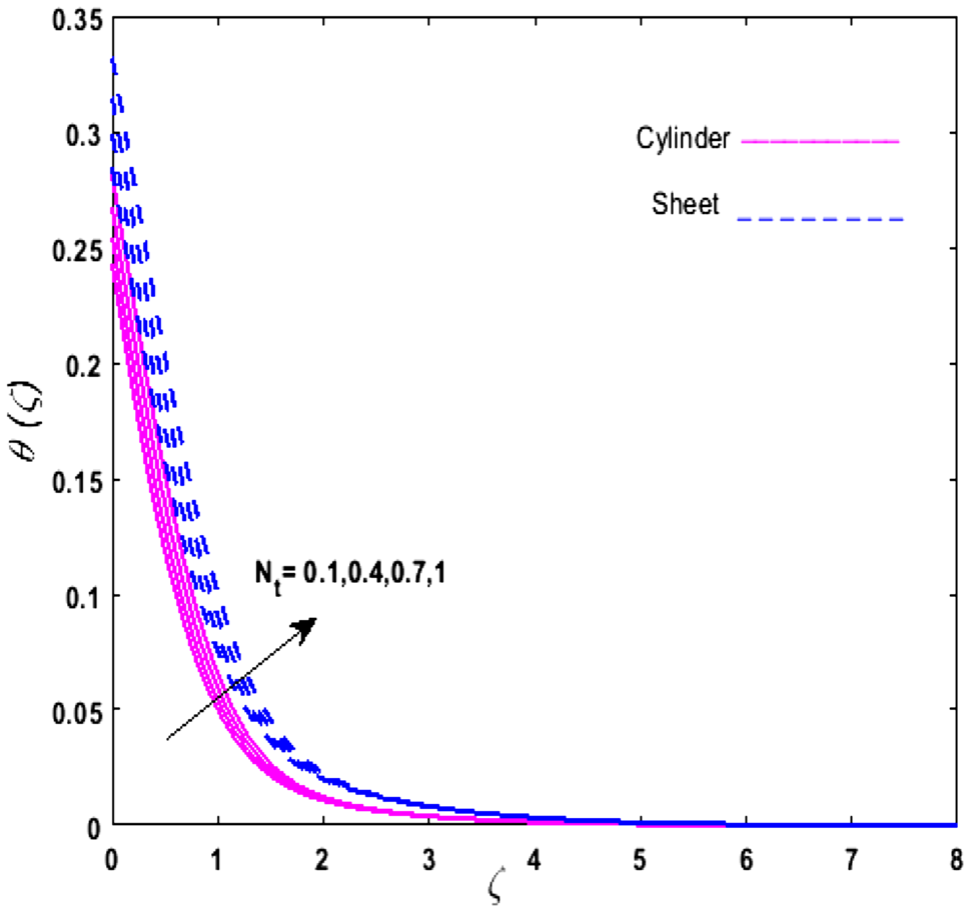
Upshot of $N_{t}$ on $\theta \left( \zeta \right)$.

**Figure 9:**
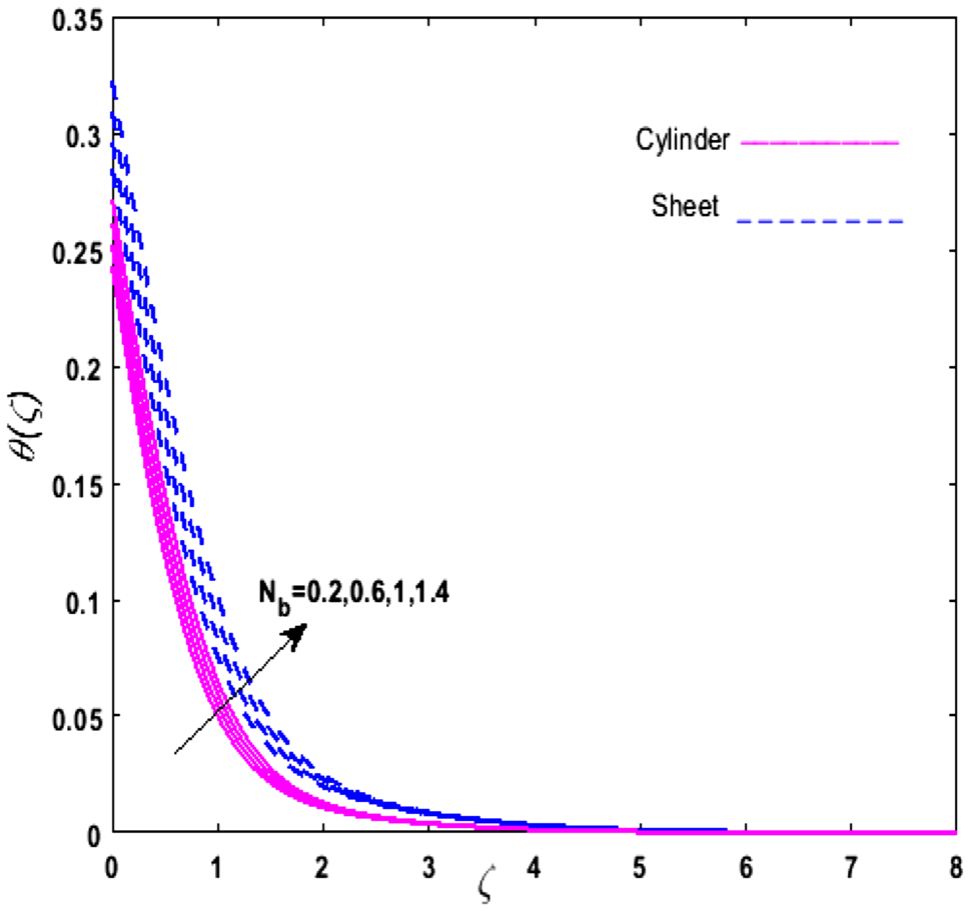
Upshot of $N_{b}$ on $\theta \left( \zeta \right)$.

**Figure 10:**
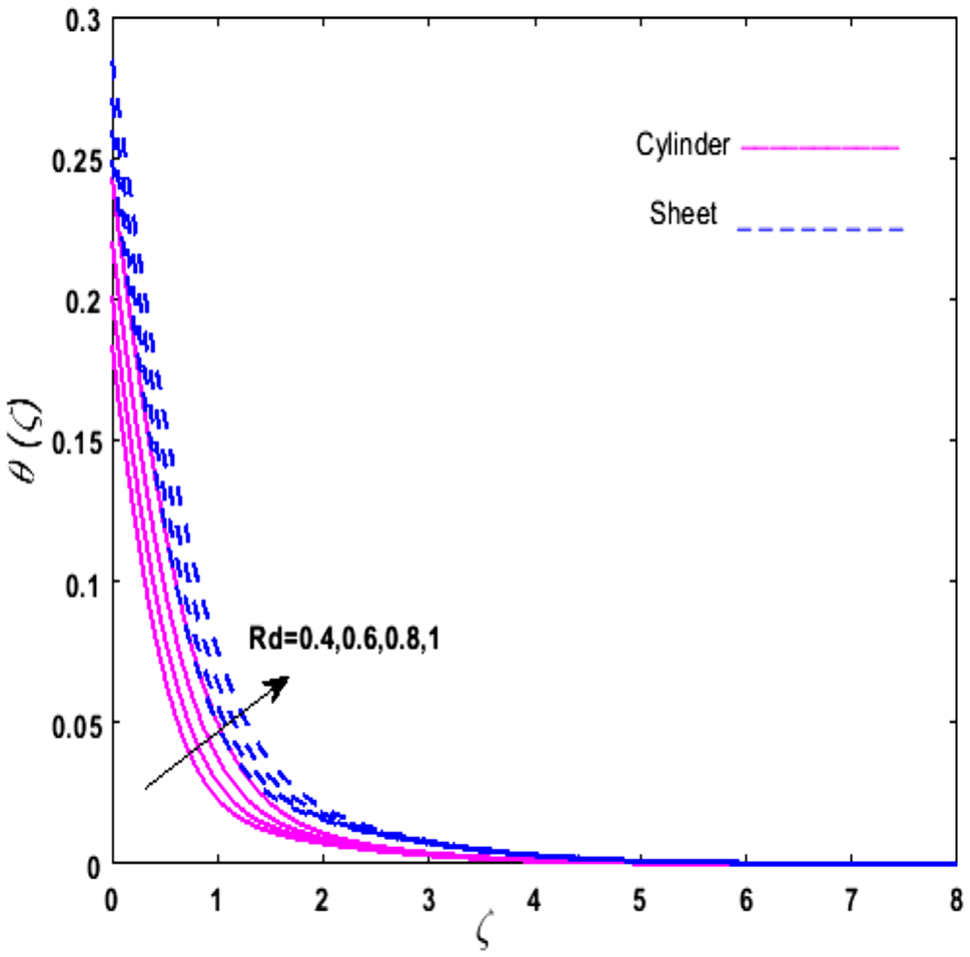
Upshot of $Rd$ on $\theta \left( \zeta \right)$.

**Figure 11:**
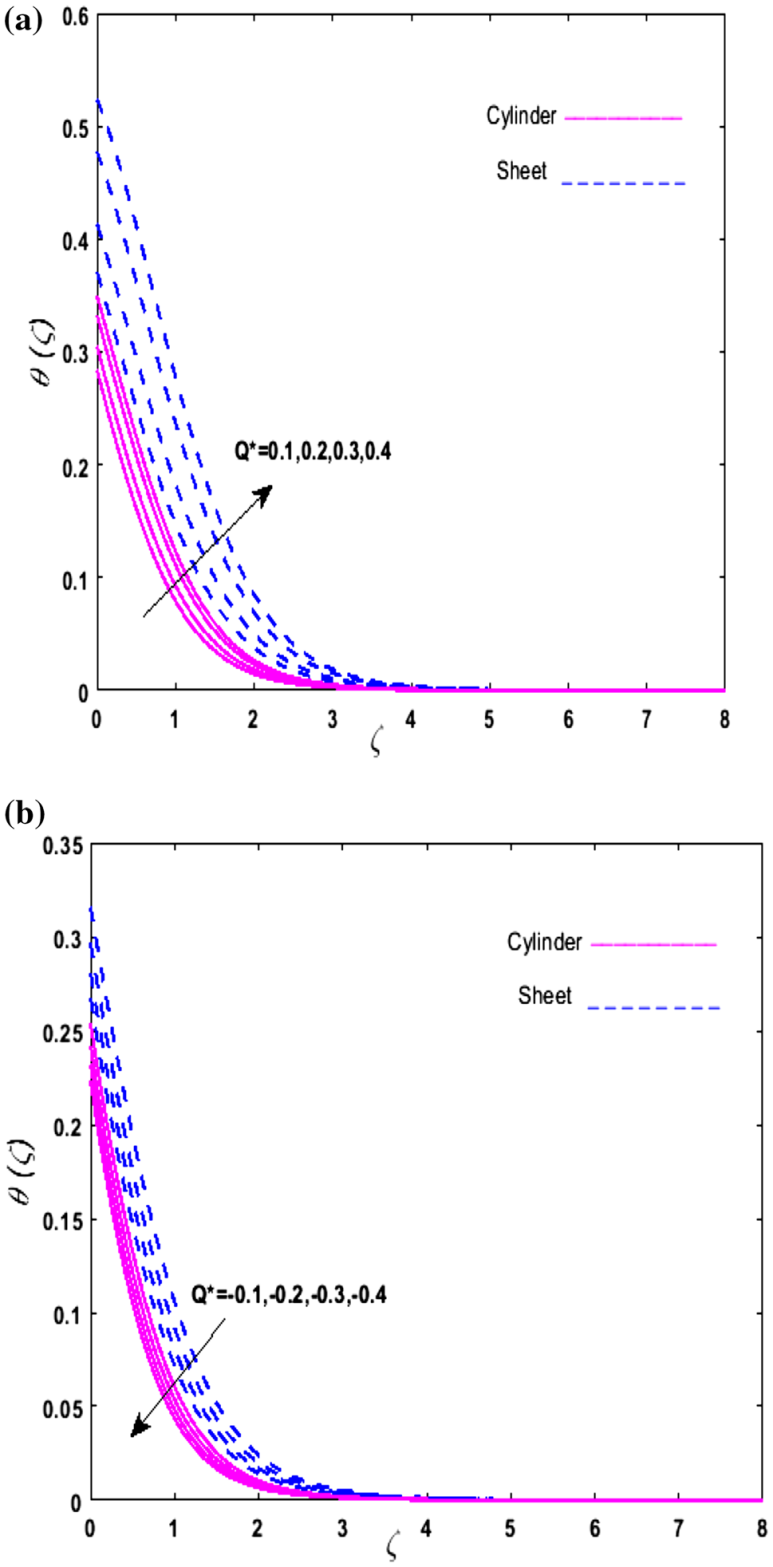
Upshot of (**a**) $\left( {Q* > 0} \right)$ on $\theta \left( \zeta \right)$, (**b**) $\left( {Q* < 0} \right)$ on $\theta \left( \zeta \right)$.

**Figure 12:**
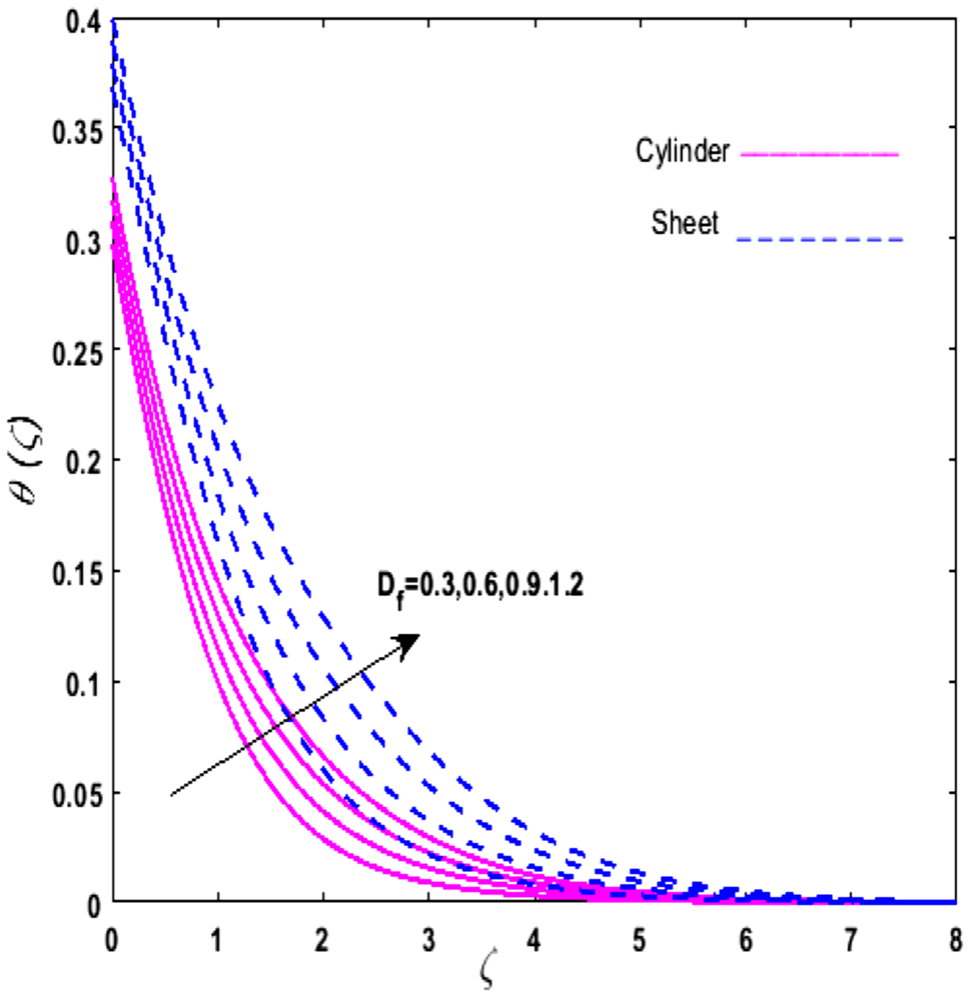
Upshot of $D_{f}$ on $\theta \left( \zeta \right)$.

**Figure 13:**
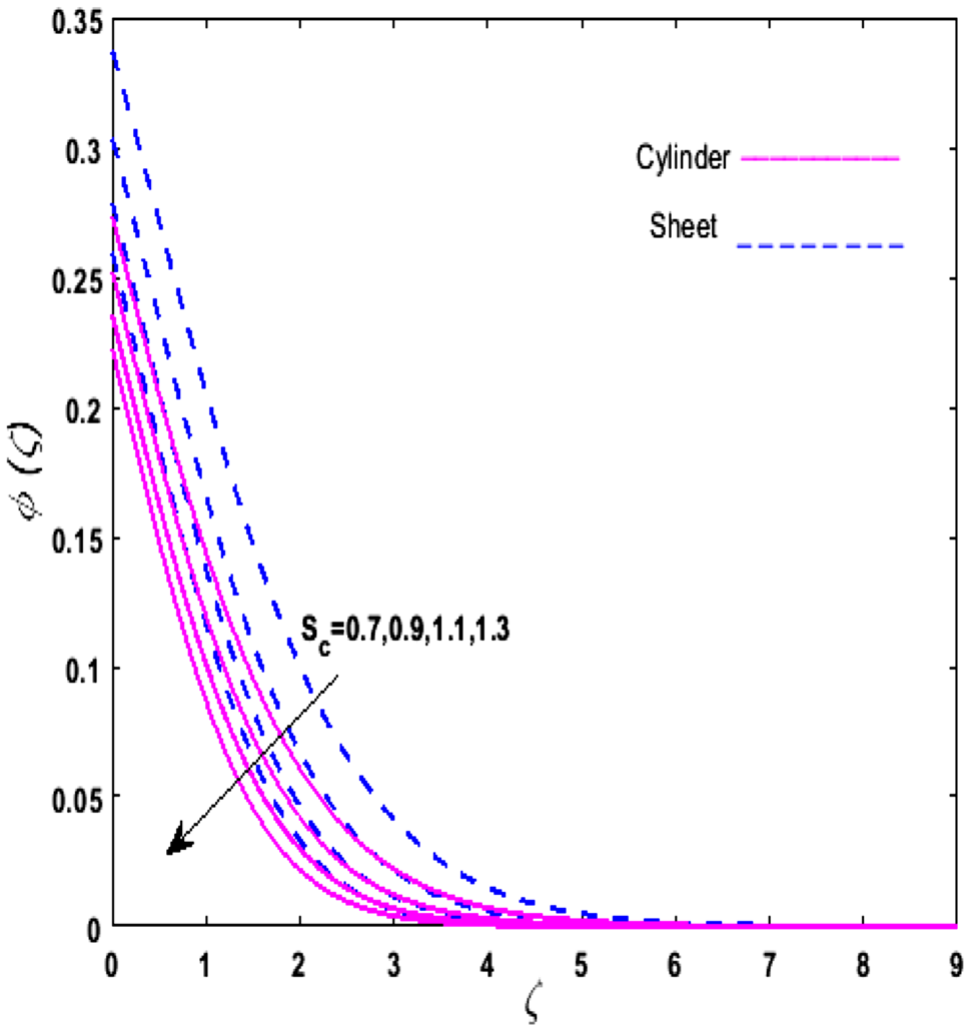
Upshot of $S_{c}$ on $\phi \left( \zeta \right)$.

**Figure 14:**
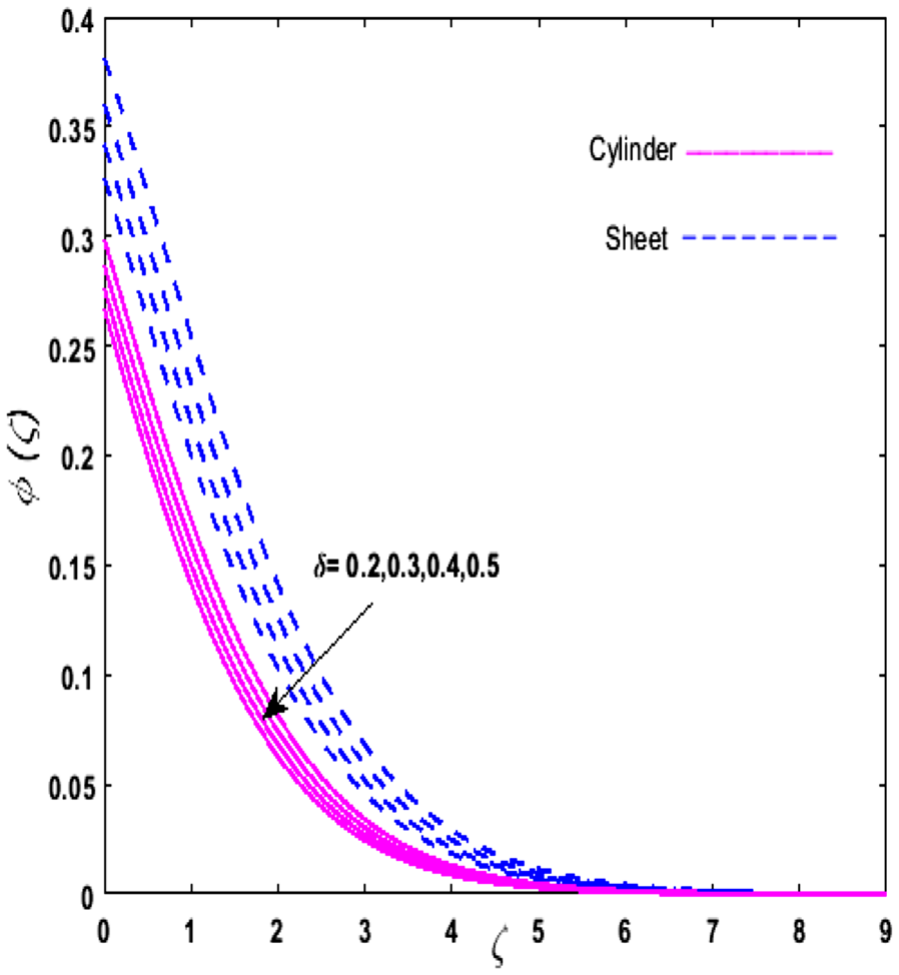
Upshot of $\delta$ on $\phi \left( \zeta \right)$.

**Figure 15:**
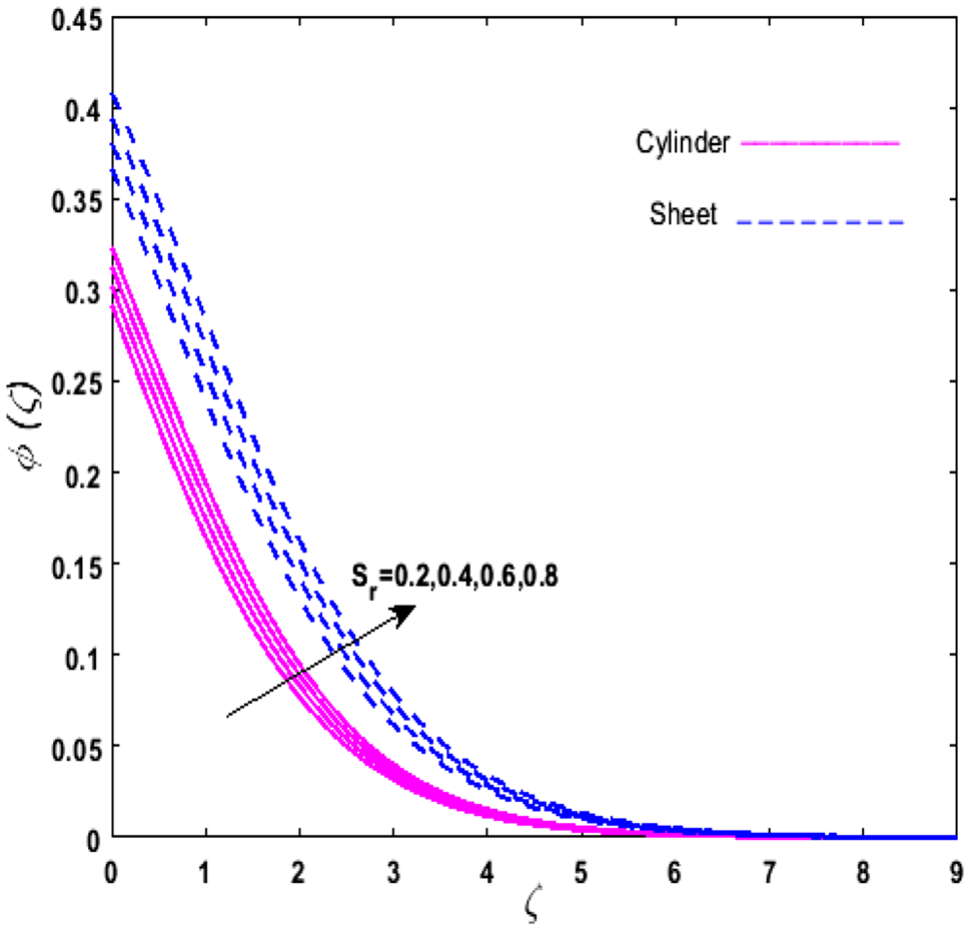
Upshot of $S_{r}$ on $\phi \left( \zeta \right)$.

**Figure 16:**
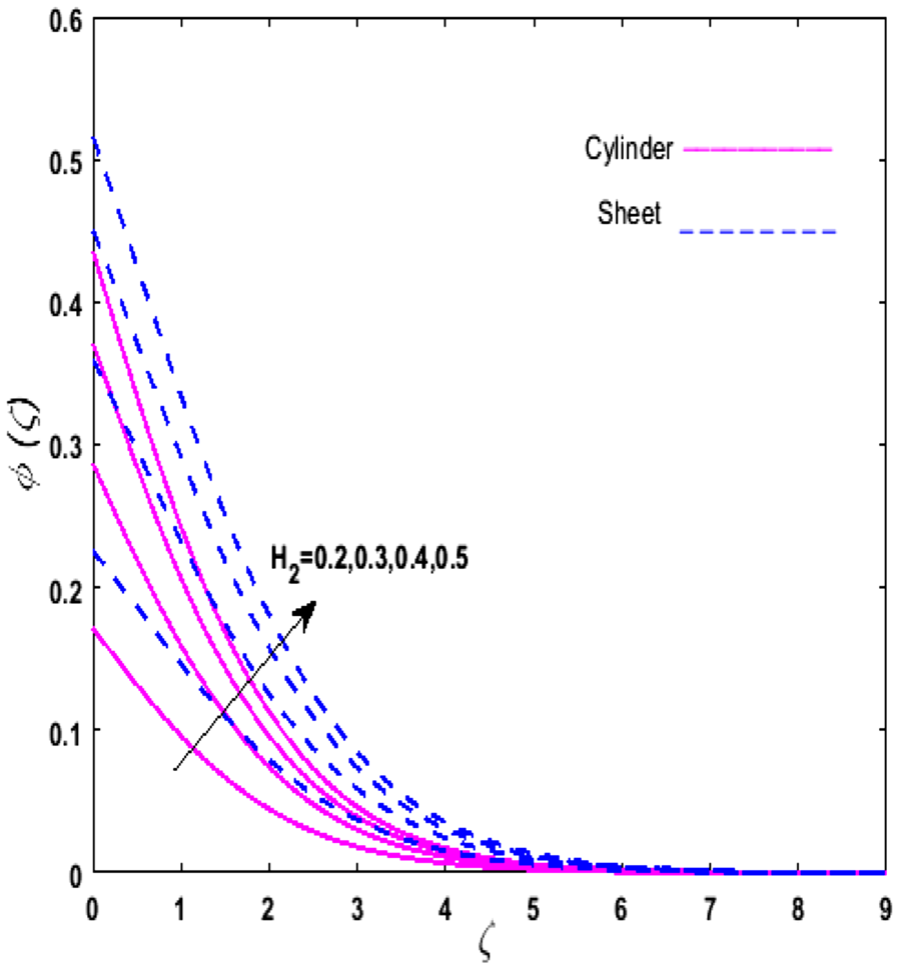
Upshot of $H_{2}$ on $\phi \left( \zeta \right)$.

**Figure 17:**
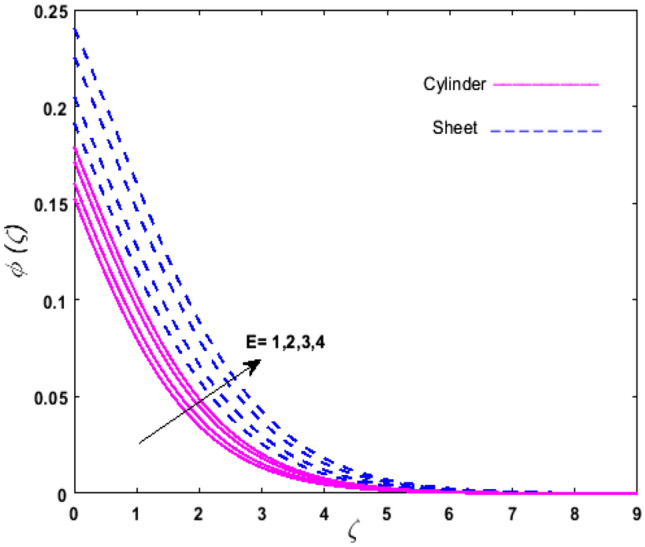
Upshot of $E$ on $\phi \left( \zeta \right)$.

For the graphical analysis of the dimensionless parameters versus involved profiles appearing in the highly nonlinear mathematical problem in Eqs. (12)–(15). This problem is elucidated numerically by utilizing bvp4c, an implemented function in MATLAB. The impression of sundry on the velocity of the fluid, transmission of heat, and mass are shown graphically in such a manner that solid lines correspond to a deformable cylinder and dotted lines for the case of deformable surface which are portrayed in Figs. 2, 3, 4, 5, 6, 7, 8, 9, 10, 11, 12, 13, 14, 15, 16 and 17. Figures 2, 3, 4, 5 and 6 demonstrate the influence of the Casson fluid parameter $\beta$, velocity slip parameter $L,$ porosity parameter $\lambda$, curvature parameter $\omega$, and magnetic parameter $Ha$ on the velocity of the fluid $f^{\prime}\left( \zeta \right)$. The aftermath of $\beta$ on velocity field is illustrated in Fig. 2. As $\beta$ is inversely proportional to yield stress $S_{y}$. It is found that on escalating $\beta$ yield stress decreases. This generates a resistive force that causes hindrance to the fluid flow. Consequently, a decreasing trend is perceived in the $f^{\prime}\left( \zeta \right)$ for both the stretchable cylinder and deforming sheet. The variation of the porosity parameter $\lambda$ on the fluid flow is presented in Fig. 3. Since $\lambda$ is the quotient of kinematic viscosity to the permeability of the porous medium. Growing values of $\lambda$ escalates the kinematic viscosity of the fluid. This accelerates the resistance in the system. It is found that on elevating $\lambda$ a deterrence is witnessed to the motion of the fluid. Due to mounting values of $\lambda$, sponginess in the medium reduces. Hence, $f^{\prime}\left( \zeta \right)$ diminishes for both the cylinder and the sheet. The impact of the velocity slip parameter $L$ on $f^{\prime}\left( \zeta \right)$ is sketched in Fig. 4. As growing values of $L$ strengthens the friction force. Thus, more liquid slips past the deforming cylinder. Therefore, fluid velocity depreciates in both cases for rising values of $L$. Hence, the behavior of the curvature parameter $\omega$ on the fluid flow is represented in Fig. 5. It is noticed that on uplifting the $\omega$, the radius of the stretching cylinder depreciates. Thus, resistivity in the fluid accelerates near the surface, whereas, far away from the stretching cylinder deterrence in the fluid declines. Therefore, $f^{\prime}\left( \zeta \right)$ augments. Figure 6 reflects the comportment of magnetic parameter $Ha$ on $f^{\prime}\left( \zeta \right)$. Rising values of $Ha$ strengthens the Lorentz force. Due to which resistance arises between the fluid and the surface. Consequently, flow over the deformable cylinder slows down. Therefore, a downfall is noticed in $f^{\prime}\left( \zeta \right)$ on enhancing the magnetic parameter. The outcome of the heat transfer Biot number $H_{1}$ on the temperature field $\theta \left( \zeta \right)$ is revealed in Fig. 7. Heat transfer coefficient upsurges on uplifting $H_{1}$. Due to which more heat is transmitted from the heated stretchable cylinder to the liquid. Hence, $\theta \left( \zeta \right)$ boosts for both cases. A prominent difference is noticed in an upsurge of temperature for the flat surface. On the other hand, the impact of temperature has a larger effect on the deformable cylinder. To elaborate the impression of the thermophoresis parameter $N_{t}$ on $\theta \left( \zeta \right)$ is mapped in Fig. 8. It is noticed that on enhancing $N_{t}$, thermophoretic force is strengthened. As a result, fluid particles move from the heated liquid to the cold fluid. Hence, enhancement in $\theta \left( \zeta \right)$ is prominent for both the deformable cylinder and the flat sheet. Figure 9 illustrates the impression of the Brownian motion parameter $N_{b}$ on $\theta \left( \zeta \right)$. For growing values of $N_{b}$ collision among the fluid particles increases due to which more heat is generated. Therefore, $\theta \left( \zeta \right)$ rises. The aftermath of temperature is elevated as well as enduring for both $N_{t}$ and $N_{b}$ for deformable sheet. Figure 10 displays the outcome of the radiation parameter $Rd$ on $\theta \left( \zeta \right)$. Since $Rd = \frac{{4\overline{\sigma }T_{\infty }^{3} }}{{3\overline{k} \, k}},$ so by escalating $Rd$ the mean absorption coefficient decreases. Therefore, due to growing values of $Rd$ more heat is transmitted to the fluid. Hence, $\theta \left( \zeta \right)$ rises. However, elevation in the temperature of the fluid for the stretching surface is eminent as well as lasting. The performance of heat generation and absorption parameter $Q^{*}$ on $\theta \left( \zeta \right)$ is addressed in Fig. 11a, b. It is seen that on amplifying $Q^{*}$ huge amount of heat is generated. Thus, more heat is added to the system. Hence, the thermal field upsurges, whereas, negative values of $Q^{*}$ less amount of heat is generated. Consequently, the thermal field deteriorates. It is perceived that temperature rise is rapid for both the cylinder and the sheet. However, it is noted that the temperature of the deformable cylinder is lower than the sheet as well as enduring far away from the surface. To understand the variation of the Dufour number $D_{f}$ on $\theta \left( \zeta \right)$ Fig. 12 is plotted. On escalating $D_{f}$ concentration gradient enhances, whereas, temperature gradient decreases which results in heat transmission. Thus, a prominent upsurge is found in the thermal state of $\theta \left( \zeta \right)$ which is quite eminent for the stretching sheet. The impression of the Schmidt number $S_{c}$ on the concentration profile $\phi \left( \zeta \right)$ is portrayed in Fig. 13. As $S_{c}$ is the quotient of kinematic viscosity $\nu$ to Brownian diffusion coefficient $D_{B}$. It is noticed that mass diffusion diminishes for growing values of $S_{c}$. This results in the reduction of the concentration of the fluid. Therefore, deteriorating nature is exhibited by $\phi \left( \zeta \right)$ on boosting $S_{c}$. Figure 14 is drawn to elucidate the upshot of dimensionless chemical reaction parameter $\delta$ on $\phi \left( \zeta \right)$. On up surging $\delta ,$ chemical molecular diffusivity reduces owing to its consumption in the reaction. Hence, it is observed that on boosting $\delta$ concentration field deteriorates. It is witnessed that the aftermath of concentration on the stretchable cylinder is much lower and long-lasting on escalating $S_{c}$ and $\delta$. Figure 15 is sketched to analyze the effect of Soret number $S_{r}$ on $\phi \left( \zeta \right)$. $S_{r}$ is the quotient of temperature difference to concentration. On escalating $S_{r}$, the temperature gradient rises. It is perceived that molecular diffusion increases. Thus, the rate of mass transfer intensifies for growing values of $S_{r}$. Consequently, $\phi \left( \zeta \right)$ enhances. Figure 16 is plotted to understand the features of mass transfer Biot number $H_{2}$. It is perceived that $H_{2}$ depends on the mass transfer coefficient $h_{2}$. Therefore, growing values of $H_{2}$ elevates $\phi \left( \zeta \right)$. The impression of mounting values of activation energy $E$ is deliberated in Fig. 17. It is noticed that escalating values of $E$ lead to a decrease in the Arrhenius function. Consequently, the generative chemical reaction decelerates. Thus, on escalating $E$, the fluid concentration upsurges. It is found that concentration distribution on augmenting $S_{r}$, $H_{2}$ and $E$ is quite eminent for sheet in comparison to the deformable cylinder. The outcome of tabulated values of dimensionless parameters $\omega ,\lambda ,Ha\,{\text{and}}\,L.$ on drag force coefficient is depicted in Table 2. It is perceived that on escalating $\omega ,\lambda \,{\text{and}}\,Ha$ shear stress increases, however, a reverse upshot is seen on mounting $L.$ The influence of $\Pr ,H_{1} ,D_{f} ,H_{2} ,$ $S_{r} ,N_{b} ,$ $S_{c} \,{\text{and}}\,N_{t}$ on local Nusselt number and Sherwood number is portrayed in Table 3. It is perceived that on escalating $D_{f} ,H_{2} ,S_{r} ,N_{b} ,S_{c} \,{\text{and}}\,N_{t}$ mass flux augments, whereas, heat flux diminishes. A deteriorating nature is exhibited by mass transfer on amplifying $\Pr {\text{ and }}H_{1}$, however, the rate of heat transfer amplifies. A comparative analysis of the present investigation is exhibited in Table 4 with Fathizadeh et al.^72^, Fang et al.^73^, and Imtiaz et al.^74^. A good association between the results is seen.

**Table 2:** Computational values of friction drag coefficient for distinct values of $\omega ,\lambda ,Ha{\text{ and }}L.$

$\omega$	$\lambda$	$Ha$	$L$	$- \left( {1 + \frac{1}{\beta }} \right)\left. {\frac{{d^{2} f}}{{d\zeta^{2} }}} \right|_{\zeta = 0}$
0.1				1.4510035
0.2				1.6070220
0.3				1.7807451
	0.4			1.5589928
	0.5			1.6088920
	0.6			1.6564151
		0.3		1.3923086
		0.5		1.5064596
		0.7		1.6088920
			0.3	1.0010071
			0.4	0.9841995
			0.5	0.8913582

**Table 3:** Computational values of $Nu_{z} \left( {{\text{Re}}_{z} } \right)^{ - 0.5}$ and $Sh_{z} \left( {{\text{Re}}_{z} } \right)^{ - 0.5}$ against the different estimation of $\Pr ,H_{1} ,D_{f} ,H_{2} ,S_{r} ,N_{b} ,S_{c} {\text{ and }}N_{t} .$

$\Pr$	$H_{1}$	$D_{f}$	$H_{2}$	$S_{r}$	$N_{b}$	$S_{c}$	$N_{t}$	$Nu_{z} \left( {{\text{Re}}_{z} } \right)^{ - 0.5}$	$Sh_{z} \left( {{\text{Re}}_{z} } \right)^{ - 0.5}$
4								0.4647132	0.07975943
6								0.4833629	0.07957307
8								0.4955689	0.07944563
	0.1							0.1441683	0.08233553
	0.3							0.3834158	0.08041072
	0.5							0.5727355	0.07880583
		0.3						0.4911270	0.07960337
		0.5						0.4812924	0.07966677
		0.7						0.4710714	0.07973468
			0.1					0.4959289	0.07957307
			0.3					0.4874835	0.17771861
			0.5					0.4828872	0.23514202
				0.2				0.4900239	0.06330082
				0.4				0.4766769	0.06918812
				0.6				0.4602900	0.07594917
					0.1			0.4978845	0.07957307
					0.3			0.4939279	0.07962442
					0.5			0.4897862	0.07967779
						0.6		0.4986354	0.07806048
						0.8		0.4959289	0.07957309
						1		0.4932983	0.08065435
							0.2	0.4969961	0.07958369
							0.4	0.4951655	0.079610632
							0.6	0.4932593	0.079636848

**Table 4:** Comparison of $f^{\prime\prime}(0)$ when $\omega = \lambda = \Pr = d = Rd$ $= N_{t} = N_{b} = Q^{*} = D_{f} = e = S_{c} = \delta$ $= \alpha = E = L = H_{1} = H_{2} = 0,\beta \to \infty$ with Fathizadeh et al.^72^, Fang et al.^73^, and Imtiaz et al.^74^ with the present work.

$Ha$	Present	Fathizadeh^72^			Fang^73^	Imtiaz^74^
	bvp4c	HPM	M-HPM	Exact solution	Exact solution	HAM
0	− 1	− 1	− 1	− 1	− 1	− 1
0.5	− 1.11825	–	–	–	− 1.1180	− 1.1180
1	− 1.41421	− 1.41421	− 1.41421	− 1.41421	–	− 1.4142
5	− 2.44943	− 2.44948	− 2.44948	− 2.44948	–	− 2.4494
10	− 3.31626	− 3.31662	− 3.31662	− 3.31662	–	–

## Concluding remarks

The numerical solution for radiative Casson nanofluid flow with variable characteristics incorporated with chemical reaction and Arrhenius activation energy has been obtained past a deformable cylinder. Transfer of heat and mass is enhanced by inspecting the impression of the Soret-Dufour factor with robin conditions. The mathematical model is deciphered through bvp4c, an implemented function in MATLAB. Graphical impressions of the parameters involved in the mathematical problem are illustrated for the deformable cylinder and stretching sheet. The perceptible analyses of the present investigation are:

- A decreasing trend is noticed in the velocity field for fluctuation in the Casson fluid parameter, velocity slip parameter, and porosity parameter.
- The thermal field amplifies escalating $Rd$.
- For growing values of $N_{t} \,{\text{and}}\,N_{b}$ temperature field augments.
- On elevating the heat transfer Biot number a prominent difference is noticed in the upsurge of temperature for the flat sheet.
- For larger values of $S_{c} \,{\text{and}}\,\delta$ the concentration field declines.
- The outcome of augmenting $S_{r}$, $H_{2}$ and $E$ is quite eminent on the concentration distribution for the sheet in comparison to the deformable cylinder.
- Drag force coefficient increases on escalating $\omega ,\lambda \,{\text{and}}\,Ha$
- The mass transfer exhibits a deteriorating impact on amplifying $\Pr \,{\text{and}}\,H_{1}$ , however, the rate of heat transfer amplifies.
- Mass flux augments on escalating $D_{f} ,N_{b} ,S_{c} \,{\text{and}}\,N_{t}$.

## List of symbols

$B_{0}$: Magnetic field strength, A m^−1^
$\tilde{C}$: Concentration of nanoparticles, kg m^−3^
$\tilde{C}_{w}$: Concentration at the surface of the cylinder
$$\tilde{C}_{\infty }$$: Ambient concentration
$$c_{s}$$: Concentration susceptibility
$$c_{p}$$: Specific heat, m^2^ s ^−2^ k^−1^
$C_{f}$: Skin friction coefficient
$D_{B}$: Brownian diffusion coefficient
$D_{T}$: Thermophoretic diffusion coefficient
$$D_{f} = \frac{{D_{T} k_{t}^{*} \left( {\tilde{C}_{w} - \tilde{C}_{\infty } } \right)}}{{\nu c_{s} c_{p} \left( {\tilde{T}_{w} - \tilde{T}_{\infty } } \right)}}$$: Dufour number
$D_{B} \left( C \right)$: Variable molecular diffusivity
$$d$$: Small parameter
$$E_{a}$$: Activation energy
$E = \frac{{E_{a} }}{{k\tilde{T}_{\infty } }}$: Activation energy parameter
$$e$$: Small parameter
$$Ha = \frac{{\sigma_{1} B_{0}^{2} l}}{{\rho U_{1} }}$$: Magnetic parameter
$h_{1}$: Convective heat transfer coefficient
$h_{2}$: Convective mass transfer coefficient
$H_{1} = \frac{{h_{1} }}{{k_{\infty } }}\sqrt {\frac{\nu l}{{U_{1} }}}$: Heat transfer Biot number
$H_{2} = \frac{{h_{2} }}{{D_{{B_{\infty } }} }}\sqrt {\frac{\nu l}{{U_{1} }}}$: Mass transfer Biot number
$K^{*}$: Permeability of porous medium
$k_{t}^{*}$: Thermal diffusion ratio
$k\left( T \right)$: Temperature-dependent thermal conductivity, W m^−1^ K^−1^
$k_{r}^{2}$: Chemical reaction parameter
$\overline{k}$: Mean absorption coefficient
$L = S\sqrt {\frac{{U_{1} }}{\nu l}}$: Velocity slip parameter
$$l$$: Characteristic length
$N_{b} = \frac{{\tau D_{B} \left( {\tilde{C}_{w} - \tilde{C}_{\infty } } \right)}}{\nu }$: Brownian motion parameter
$$N_{t} = \frac{{\tau D_{T} \left( {\tilde{T}_{w} - \tilde{T}_{\infty } } \right)}}{{\nu \tilde{T}_{\infty } }}$$: Thermophoretic parameter
$Nu_{z}$: Local Nusselt number
$$\Pr = \frac{{\mu c_{p} }}{{k_{\infty } }}$$: Prandtl number
$Q_{1}$: Coefficient of heat generated or absorbed per unit volume
$q_{r}$: Radiative heat flux
$Q^{*} = \frac{{Q_{1} l}}{{\rho c_{p} U_{1} }}$: Heat generation/absorption parameter
$$Q_{w}$$: Heat flux
$$Q_{m}$$: Mass flux
${\text{Re}}_{z} = \frac{{U_{1} z^{2} }}{\nu l}$: Local Reynold number
$$Rd = \frac{{4\overline{\sigma }T_{\infty }^{3} }}{{\overline{k}k_{\infty } }}$$: Radiation parameter
$S$: Velocity slip factor
$Sh_{z}$: Local Sherwood number
$$S_{c} = \frac{\nu }{{D_{B} }}$$: Schmidt number
$$S_{r} = \frac{{D_{T} k_{t}^{*} \left( {\tilde{T}_{w} - \tilde{T}_{\infty } } \right)}}{{\nu \tilde{T}_{\infty } \left( {\tilde{C}_{w} - \tilde{C}_{\infty } } \right)}}$$: Soret number
$Sh_{z}$: Local Sherwood number
$S$: Velocity slip factor
$$\tilde{T}$$: Temperature, K
$\tilde{T}_{w}$: Temperature at the surface of the cylinder, K
$$\tilde{T}_{\infty }$$: Ambient temperature, K
$\tilde{u},\tilde{w}$: Component of velocity in the direction of $r\& z$
$$U_{1}$$: Reference velocity
$r,z$: Cylindrical polar coordinates
*r*-axis: Radial direction
*z*-axis: Axial direction
$$W_{w} = \frac{{U_{1} z}}{l}$$: Velocity (stretching), m s^−1^

## Greek symbols

$$\nu$$: Kinematic viscosity, m^2^ s^−1^
$\mu$: Dynamic viscosity, Pa s
$\overline{\sigma }$: Stefan-Boltzmann coefficient, eVk^−1^
$\sigma_{1}$: Electrical conductivity, s^3^ m^2^ kg^−1^
$\rho$: Fluid density, kg m^−3^
$$\lambda = \frac{\nu l}{{K^{*} U_{1} }}$$: Porosity parameter
$\delta = \frac{{k_{r}^{2} l}}{{U_{1} }}$: Chemical reaction parameter
$$\alpha = \frac{{\tilde{T}_{w} - \tilde{T}_{\infty } }}{{\tilde{T}_{\infty } }}$$: Temperature difference
$$\omega = \sqrt {\frac{\nu l}{{U_{1} R^{2} }}}$$: Curvature parameter
$$\beta = \frac{{\mu_{c} \sqrt {2\Pi_{c} } }}{{S_{y} }}$$: Casson parameter
$$\zeta$$: Dimensionless parameter
$\tau_{w}$: Shear stress at the surface
